# Psychopathological Symptoms under Smog: The Role of Emotion Regulation

**DOI:** 10.3389/fpsyg.2017.02274

**Published:** 2018-01-17

**Authors:** Shuquan Chen, Jiayang Kong, Feng Yu, Kaiping Peng

**Affiliations:** ^1^Department of Psychology, School of Social Sciences, Tsinghua University, Beijing, China; ^2^School of Life Sciences, Tsinghua University, Beijing, China; ^3^School of Humanity and Social Sciences, Xi'an Jiaotong University, Xian, China

**Keywords:** air pollution, smog, psychopathology, emotion regulation, context

## Abstract

Over the past decade, major cities in China have suffered from severe air pollution, which is also known as smog. Despite lay considerations that smog might pose risks for psychopathology, it remains unknown whether it is only linked to affective psychopathology or to a broader range of symptomologies. Moreover, whether individual differences in emotion regulation, a transdiagnostic risk factor for psychopathology, would influence the magnitude of pollution-induced symptoms is not well understood. Using a longitudinal design, the current study measured trait emotion regulation and psychopathological symptoms in a sample of university students at Time 1 (without smog, *N* = 120) and then reassessed for psychopathology at Time 2 (after acute exposure to smog for 1 week, *N* = 102). The results showed that participants had higher levels of positive symptom distress, obsessive-compulsive symptoms, interpersonal sensitivity, depression, and psychoticism at Time 2. Moreover, reappraisal is negatively associated with smog-induced elevations in psychopathological symptoms only when participants rely heavily on suppression. We discuss the implications of this investigation for both intervention efforts and future work on the contextual factors surrounding the deployment of emotion regulation strategies.

## Introduction

Severe air pollution in China has been referred as smog and become a critical problem influencing millions of people. According to Schwarze et al. (2006), air pollution is a toxic and complex mixture of pollutants including particulate matter (PM), chemical substances and biological materials. All the aforementioned components are deleterious to human health, but the most severe effects are attributed to ambient PM, the damaging potential of which is inversely related to its aerodynamic diameter (Schwarze et al., 2006; Franck et al., 2011; Franchini and Mannucci, 2015). As proposed by World Health Organization (WHO, 2006), the air quality guidelines for the 24-h mean value of PM smaller than 2.5 μm (PM_2.5_) and PM smaller than 10 μm (PM_10_) are respectively 25 and 50 μg/m^3^, but both PM_2.5_ and PM_10_ at major cities in China (e.g., Beijing) have far exceeded these numbers in winter and spring for the past decade (Chen et al., 2017).

Under such pressing circumstances, there is an increasing awareness that acute and chronic exposure to smog might have a wide range of detrimental effects on human health. It is true that previous findings have established links between air pollution and several physical diseases (Arbex et al., 2009; Pope et al., 2011; Zhang et al., 2011; Chen et al., 2013; Baumgartner et al., 2014), but relatively few existing literature has investigated the link between smog exposure in China and mental health (Zhang et al., 2017). Whether smog induces a wide range of psychopathological symptoms or just a relatively small repertoire of symptoms such as depression remains unknown. Additionally, whether certain individual differences could alter psychopathological symptoms following acute exposure to smog, is not well understood. Both of the questions mentioned above, however, are of great importance if we were to develop potential intervention options for smog-induced psychopathology.

### Air pollution and physical health

The deleterious effects of air pollution on physical disease have been examined extensively in existing literature. For instance, air pollution is associated with chronic obstructive pulmonary disease (Arbex et al., 2009), higher blood pressure (Baumgartner et al., 2014), cardiovascular and cerebrovascular disease (Zhang et al., 2011) and lung cancer (Pope et al., 2011). More strikingly, using a quasi-experimental empirical approach, Chen et al. (2013) found that ambient concentrations of total suspended particulates are about 55% higher in northern China due to winter heating; as a result, incidence of cardiorespiratory mortality is significantly higher in the north leading to 5.5 years lower of life expectancies for northern citizens. This is in accordance with the finding by Zhang et al. (2011) that long-term exposure to ambient air pollution is associated with mortality due to cardiovascular and cerebrovascular diseases. These authors drew the conclusions by taking into account the time course of air pollution, cardiovascular as well as cerebrovascular diseases and mortality. Considering that smog in China is an extremely severe case of air pollution with PM exceeding 10 times of the number proposed by air quality guidelines, it is not surprising that some of the aforementioned findings are based on studies using Chinese database and samples.

### Air pollution and psychological health

In spite of the great attention paid to air pollution and its influences on physical health, the impact of air pollution on psychological health is less frequently investigated thereby remaining unclear. To date, some studies in western societies have suggested that toxic air pollution leads to higher level of psychopathological symptoms especially externalizing psychopathology (for a review, see Bell, 1993). It is very important to keep in mind, nevertheless, that most of the findings cited in the review relied on correlational analysis using epidemiological data in several consecutive years, making it impossible to determine whether air pollution is indeed causally linked to psychopathology. For instance, it is probable that a third variable, such as increased unemployment rate due to the promotion of large-scale machine industry, is playing a role. Moreover, the nature of air pollution is not necessarily the same in China and western countries given that the major pollutants in China are PM. As such, it seems critically important to test the causal relationship between air pollution and psychopathology in China.

In lay options, severe air pollution in China is thought to endanger not only physical but also psychological health. Nevertheless, relatively few empirical studies examine such an association. One notable exception, a longitudinal nationwide study, found that air pollution reduced hedonic happiness and increased the rate of depressive symptoms (Zhang et al., 2017). This study provides us with the first empirical evidence linking air pollution in China to depression, yet there are still at least two questions that remain unclear.

First, whether air pollution is uniquely associated with depression or a broader range of symptomologies is still unknown. It is possible that acute exposure to air pollution would lead to an increase in not only depressive symptoms but also other symptomologies such as anxiety and obsessive-compulsive symptoms. Indeed, it is possible that the influence of air pollution is not limited to depression considering the large amount of comorbidity between depression and other disorders (Kessler et al., 2005). However, given the discrepancies over the cause of psychopathology, symptoms induced by smog might not necessarily share the same mechanisms as those examined in previous literature. As such, it would be beneficial to assess a wide range of psychopathological symptoms to see if pollution induces a wide range of psychopathology or just depression.

Second, living in smog is a significant day-to-day stressor evoking negative emotions (Zhang et al., 2017). Under such circumstances, effective self-regulation of affect might be critical to cope with such a stressor and especially important for individuals faced with severe air pollution. Indeed, the experience of negative events plays a critical role in the onset of psychopathology especially depression (Brown and Harris, 2012), but only a minority of those who experience negative life events would develop a full-blown depressive episode. It has been suggested that self-regulation of emotion might play an important role in disordered affect following negative life events (for a review, see Joormann and Stanton, 2016). However, to date no study has examined whether individual differences in self-regulation of affect would be a factor influencing the development of pollution-induced psychopathology. Why some people's affect recovers easily even when exposed to smog whereas other people struggle in the same situation is a fascinating question with important implications for the onset and possible prevention of not only pollution-induced depression but also other forms of psychopathology. Therefore, it would be informative to examine the link between use frequencies of emotion regulation strategies and psychopathological symptoms following acute exposure to air pollution.

### Possible effects of cognitive reappraisal and expressive suppression on smog-induced symptoms

Given the critical role of emotion regulation in the etiology of psychopathology (Aldao and Nolen-Hoeksema, 2012; Joormann and Stanton, 2016), it is likely that more frequent use of adaptive strategies and less frequent use of maladaptive strategies is linked to less psychopathological symptoms following exposure to smog.

The two most frequently examined strategies, cognitive reappraisal and expressive suppression, have been shown as transdiagnostic factors for psychopathology (Aldao et al., 2010; Webb et al., 2012). Reappraisal refers to changing a situation's meaning so as to alter one's emotional response to the situation (Gross and John, 2003). Several meta-analysis articles suggest a beneficial effect of reappraisal on mental health, but it should be noted that the effect size is relatively small (Aldao et al., 2010; Webb et al., 2012). Moreover, other authors have found that reappraisal can be ineffective when used in the wrong context (Troy et al., 2013). These authors showed that reappraisal is adaptive when using in face with uncontrollable stressful life events while maladaptive when using in relatively controllable stressors. Similarly, using both self-report and experimental assessment of reappraisal, Troy et al. (2017) found that effectively regulating one's emotions using cognitive reappraisal could be more beneficial in lower vs. social economic status contexts. These authors further discussed this finding by suggesting that people with lower social economic status might have less resources to control their surroundings thereby leaving them no option but to self-regulate. Being exposed to air pollution is equal and almost unchangeable for people living in cities with air pollution. Considering this relatively uncontrollable nature of severe air pollution, it is highly possible that higher use frequency of reappraisal would buffer the undesirable impact of air pollution on psychopathology.

Suppression, on the other hand, involves attempts to inhibit the effects of external cues on internal and external states of emotion (Gross and John, 2003). Whereas some researchers suggest that it is maladaptive (Gross and John, 2003), other researchers find no such link (D'Avanzato et al., 2013), or even beneficial effect of suppression on reducing psychological distress following extreme life events (Dunn et al., 2009). These mixed findings suggest that empirical study is needed if we were to determine the adaptiveness of suppression in face with smog.

On the surface, it seems as if reappraisal and suppression work separately, but in fact they are largely interactive. For instance, Aldao and Nolen-Hoeksema (2012) found that the composite score of the use frequencies of several adaptive strategies is negatively associated with psychopathological symptoms only when the composite score of the use frequencies of several maladaptive strategies is high. The authors further discussed this result and suggested that adaptive strategies play its protective role by functioning as a buffer to the harmful effects of maladaptive strategies. It is important to keep in mind, therefore, reappraisal and suppression might interact to predict pollution-induced symptoms in addition to their independent effects. To be specific, it is hypothesized in the present study that reappraisal is more adaptive when the use frequencies of suppression is high for the individuals.

### The present study

In the present study, we followed 120 university students at a 1 month interval and measured their symptoms of psychopathology at the two time points. The first time point was at the end of a week that is relatively free of pollution, while the second time point followed a severely polluted week. By such longitudinal design, we hope to compare symptoms across these two conditions so as to determine if acute exposure to smog does increase psychopathological symptoms. In addition, habitual use of reappraisal and suppression, which have been identified as stable traits over time (Gross and John, 2003), is assessed during the baseline assessment so that we could test the possible effects of emotion regulation on pollution-induced symptoms.

## Methods

### Participants and procedures

One hundred and twenty undergraduate students completed online questionnaires at two time points for course credits. They were recruited from a university in Beijing. Eighteen participants only participated in the study at Time 1, which left 102 participants for the final analysis. The participants' age ranged from 17 to 22 (*M* = 18.03, *SD* = 1.03), and 58.8% of the sample were females. At Time 1, participants provided demographical information and completed instruments measuring psychopathological symptoms as well as trait emotion regulation. One month later (Time 2), psychopathological symptoms were reassessed. The Institutional Review Board of Tsinghua University approved the research protocol, and prior completing the Time 1 assessment all participants signed informed consent. After the Time 2 assessment, participants were debriefed.

The week prior to the Time 1 assessment was relatively free of air pollution, with a mean PM_2.5_ of 16.1 μg/m^3^ (average by hour). In contrast, the week prior to the Time 2 assessment was moderately to severely polluted, with a mean PM_2.5_ of 173.5 μg/m^3^, which was significantly higher than that of the week before Time 1 [*t*_(238)_ = 17.61, *p* < 0.001]. The temperature at Time 1 was similar to that at Time 2, both having an average temperature of 12°C. Moreover, the measures at Time 1 took place on a cloudy day. In this way, we managed to control both the temperature and light, which have been found to be influential factors for seasonal affective disorders (SAD; Miller, 2005).

### Measures

Psychopathology was assessed using the *Symptom Check List-90* (SCL-90; Derogatis and Cleary, 1977). The Chinese version of the SCL-90 we used was translated and published in China (Wang, 1984). The SCL-90 is comprised of 90 items, each measured on a 5-point scale of distress from “not at all” (0) to “extremely” (4). Current psychopathology is reflected in terms of nine primary symptom dimensions: somatization (SOM), obsessive-compulsive (O-C), interpersonal sensitivity (INS), depression (DEP), anxiety (ANX), hostility (HOS), phobic anxiety (PHO), paranoid ideation (PAR) and psychoticism (PSY). Moreover, scores were averaged among positive symptom dimensions to reflect the level of positive symptom distress (PSD). In this study, all subscales of SCL-90 were internally consistent (all α.s. > 0.70 in the present study).

Additionally, we used the *Emotion Regulation Questionnaire* (ERQ; Gross and John, 2003) to assess the frequency with which people use cognitive reappraisal or expressive suppression to regulate affect. The ERQ consists of two subscales that measure the habitual use of reappraisal and suppression using a 7-point likert scale. The reappraisal subscale (e.g., *I control my emotions by changing the way I think about the situation I'm in*) consists of 6 items (α = 0.85 in the present study) and the suppression subscale (e.g., *I keep my emotion to myself*) consists of 4 items (α = 0.80 in the present study). The Chinese revised version of the scale was utilized in the current study (Wang et al., 2007).

## Results

### Comparison between SCL-90 scores in days with and without air pollution

Within-group comparisons showed that participants reported significantly higher levels of obsessive-compulsive symptoms on the day with air pollution (*M* = 2.00, *SD* = 0.60), compared with the day without air pollution (*M* = 1.87, *SD* = 0.60), *t*_(101)_ = 3.11, *p* < 0.01, *d* = 0.123, 95% CI [0.04, 0.20]. Participants also reported significantly higher levels of interpersonal sensitivity on the day with air pollution (*M* = 1.83, *SD* = 0.68), compared with the day without air pollution (*M* = 1.68, *SD* = 0.69), *t*_(101)_ = 3.20, *p* < 0.01, *d* = 0.144, 95% CI [0.05, 0.23]. In addition, the depression level of participants on the day with air pollution (*M* = 1.69, *SD* = 0.66) was significantly higher than on the day without air pollution (*M* = 1.62, *SD* = 0.61), *t*_(101)_ = 2.04, *p* < 0.05, *d* = 0.076, 95% CI [0.00, 0.15]. They also reported significantly higher levels of psychoticism on the day with air pollution (*M* = 1.49, *SD* = 0.46), compared with the day without air pollution (*M* = 1.38, *SD* = 0.40), *t*_(101)_ = 3.49, *p* < 0.01, *d* = 0.112, 95% CI [0.05, 0.18]. The PSD level on the day with air pollution was also significantly higher (*M* = 2.50, *SD* = 0.45), compared with the day without air pollution (*M* = 2.43, *SD* = 0.44), *t*_(101)_ = 2.93, *p* < 0.01, *d* = 0.079, 95% CI [0.03, 0.13]. Together, the results indicate that air pollution could influence individuals' psychopathological symptoms in multiple ways (see Table 1).

**Table 1:** Comparison between days with and without air pollution.

	**Mean(*****SD*****)**		***T***	***p***	**Mean difference(95% CI)**	
	**With pollution**	**Without pollution**				
PSD Level	2.50 (0.45)	2.43 (0.44)	2.93	0.004	0.079	(0.03, 0.13)
Somatization	1.32 (0.39)	1.27 (0.33)	1.48	0.142	0.046	(−0.02, 0.11)
Obsessive-Compulsive	2.00 (0.60)	1.87 (0.60)	3.11	0.002	0.123	(0.04, 0.20)
Interpersonal Sensitivity	1.83 (0.68)	1.68 (0.69)	3.20	0.002	0.144	(0.05, 0.23)
Depression	1.69 (0.66)	1.62 (0.61)	2.04	0.044	0.076	(0.00, 0.15)
Anxiety	1.60 (0.61)	1.55 (0.58)	1.24	0.218	0.054	(−0.03, 0.14)
Hostility	1.46 (0.64)	1.44 (0.58)	0.42	0.676	0.018	(−0.07, 0.10)
Photic Anxiety	1.30 (0.42)	1.26 (0.39)	1.15	0.254	0.036	(−0.03, 0.10)
Paranoid ideation	1.55 (0.58)	1.52 (0.63)	0.99	0.325	0.036	(−0.04, 0.11)
Psychoticism	1.49 (0.46)	1.38 (0.40)	3.49	0.001	0.112	(0.05, 0.18)

*PSD, Positive Symptom Distress; CI, confidence interval*.

### Associations between SCL-90 and ERQ

We generated 10 items of change scores from SCL-90 scales by subtracting the scores on the day without air pollution from those on the day with pollution. We then executed zero-order correlations between these items (SOM change score, O-C change score, INS change score, DEP change score, anxiety change score, HOS change score, PHO change score, PAR change score, PSY change score, and PSD level change score) and another two items generated from ERQ (reappraisal and suppression) (see Table 2). The PSD level change score (*r* = −0.23, *p* < 0.05), the SOM change score (*r* = −0.38, *p* < 0.01), the INS change score (*r* = −0.29, *p* < 0.01), the DEP change score (*r* = −0.21, *p* < 0.05), the ANX change score (*r* = −0.28, *p* < 0.01) and the HOS change score (*r* = −0.22, *p* < 0.05) were negatively correlated with the suppression strategy score. However, none of the change scores was correlated with the reappraisal strategy score.

**Table 2:** Zero-order correlations between emotion regulation symptom change scores.

	**1.**	**2.**	**3.**	**4.**	**5.**	**6.**	**7.**	**8.**	**9.**	**10.**	**11.**	**12.**
1. ERQ reappraisal	–	–	–	–	–	–	–	–	–	–	–	–
2. ERQ suppression	0.15	–	–	–	–	–	–	–	–	–	–	–
3. PSD level change score	−0.07	−0.23	–	–	–	–	–	–	–	–	–	–
4. SOM change score	−0.11	−0.38	0.49	–	–	–	–	–	–	–	–	–
5. O-C change score	0.05	−0.12	0.51	0.37	–	–	–	–	–	–	–	–
6. INS change score	−0.08	−0.29	0.62	0.37	0.54	–	–	–	–	–	–	–
7. DEP change score	−0.02	−0.21	0.66	0.61	0.55	0.59	–	–	–	–	–	–
8. ANX change score	−0.09	−0.28	0.66	0.53	0.50	0.68	0.64	–	–	–	–	–
9. HOS change score	−0.13	−0.22	0.67	0.42	0.40	0.57	0.60	0.70	–	–	–	–
10. PHO change score	−0.05	−0.11	0.31	0.26	0.33	0.48	0.28	0.45	0.35	–	–	–
11. PAR change score	−0.04	−0.14	0.54	0.38	0.49	0.53	0.52	0.54	0.56	0.32	–	–
12. PSY change score	−0.02	−0.02	0.58	0.42	0.50	0.63	0.45	0.68	0.49	0.40	0.54	–

*ERQ, Emotional Regulation Questionnaire; PSD, Positive Symptom Distress; SOM, somatization; O-C, Obsessive-Compulsive; INS, interpersonal sensitivity; DEP, depression; ANX, anxiety; HOS, hostility; PHO, photic anxiety; PAR, paranoid ideation; PSY, psychoticism; Coefficients greater than 0.19 significant at p < 0.05; coefficient greater than 0.25 significant at p < 0.01*.

### Regression analyses

We predicted the PSD level change score using a hierarchical linear regression (see Table 3). In Step 1, we included gender and age variables to control gender/age-related differences. The model was insignificant, Δ*R*^2^ = 0.02, Δ*F*_(2, 99)_ = 2.20*, ns*. In Step 2, we entered suppression and reappraisal strategies scores (which were centered to reduce multicollinearity; Tabachnick and Fidell, 2007). However, they did not significantly improve the model, Δ*R*^2^ = 0.06, Δ*F*_(2, 97)_ = 2.96*, ns*. The suppression strategy score contributed significantly to the prediction of the PSD level change score, *t*_(97)_ = −2.27, *p* < 0.0*5*, β = −0.23. The reappraisal strategy score was not a significant predictor, *t*_(97)_ = −0.55, *ns*, β = −0.05. In Step 3, we added the interaction term between the reappraisal and suppression strategies, Δ*R*^2^ = 0.13, Δ*F*_(1, 96)_ = 8.88, *p* < 0.01. The suppression strategy was no longer a significant predictor, *t*_(96)_ = −1.49, *ns*, β = −0.15, and the reappraisal strategy remained an insignificant predictor, *t*_(96)_ = −0.93, *ns*, β = −0.09. However, the interaction term between the reappraisal and suppression strategies was significant, *t*_(96)_ = −2.97, *p* < 0.01, β = −0.29. We further investigated the change scores of SOM, INS, DEP, ANX, and HOS using the same method. Except for the SOM change scores, similar results were found in the final model. For INS change scores [*R*^2^ = 0.195, *F*_(5, 96)_ = 4.66, *p* < 0.01], reappraisal [*t*_(96)_ = −0.70*, ns*, β = −0.07] and suppression [*t*_(96)_ = −1.95*, ns*, β = −0.19] were insignificant while the interaction term was marginally significant, *t*_(96)_ = −1.79, *p* = 0.08, β = −0.17. For DEP change scores [*R*^2^ = 0.095, *F*_(5, 96)_ = 2.02, *ns*], reappraisal [*t*_(96)_ = −0.17, *ns*, β = −0.02] and suppression [*t*_(96)_ = −1.49*, ns*, β = −0.16] were insignificant while the interaction term was marginally significant, *t*_(96)_ = −1.94, *p* = 0.06, β = −0.20. For ANX change scores [*R*^2^ = 0.134, *F*_(5, 96)_ = 2.98, *p* < 0.05], reappraisal [*t*_(96)_ = −0.76, *ns*, β = −0.07] and suppression [*t*_(96)_ = −0.74, *ns*, β = −0.08] were insignificant while the interaction term was significant, *t*_(96)_ = −2.68*, p* < 0.01, β = −0.27. For HOS change scores [*R*^2^ = 0.074, *F*_(5, 96)_ = 1.54, *ns*], reappraisal [*t*_(96)_ = −0.54, *ns*, β = −0.05] and suppression [*t*_(96)_ = 0.44, *ns*, β = 0.05] were insignificant while the interaction term was marginally significant, *t*_(96)_ = −1.96, *p* = 0.05, β = −0.20.

**Table 3:** Hierarchical linear regression of PSD level change score With ERQ reappraisal, ERQ suppression and the interaction term between ERQ reappraisal and ERQ suppression.

**Variable**	**B**	***SE***	**95% CI**	**β**	***t***	***p***
**STEP 1**						
Gender	0.094	0.056	[−0.02, 0.21]	0.17	1.68	0.097
Age	−0.043	0.027	[−0.10, 0.01]	−0.16	−1.61	0.111
**STEP 2**						
Gender	0.112	0.056	[0.00, 0.22]	0.20	2.00	0.048
Age	−0.034	0.027	[−0.09, 0.02]	−0.13	−1.28	0.203
ERQ reappraisal	−0.015	0.027	[−0.07, 0.04]	−0.05	−0.55	0.584
ERQ suppression	−0.062	0.027	[−0.12, −0.01]	−0.23	−2.27	0.026
**STEP 3**						
Gender	0.082	0.054	[−0.03, 0.19]	0.15	1.51	0.135
Age	−0.027	0.026	[−0.08, 0.02]	−0.10	−1.03	0.304
ERQ reappraisal	−0.024	0.026	[−0.08, 0.03]	−0.09	−0.93	0.357
ERQ suppression	−0.040	0.027	[−0.09, 0.01]	−0.15	−1.49	0.138
Reappraisal × Suppression	−0.067	0.023	[−0.11, −0.02]	−0.29	−2.97	0.004
Model	Δ*R*^2^	Δ*F*	*p*			
Step 1	0.02	2.20	0.116			
Step 2	0.06	2.96	0.057			
Step 3	0.13	8.79	0.004			

*PSD, Positive Symptom Distress; ERQ, Emotional Regulation Questionnaire; CI, Confidence interval*.

**Figure 1:**
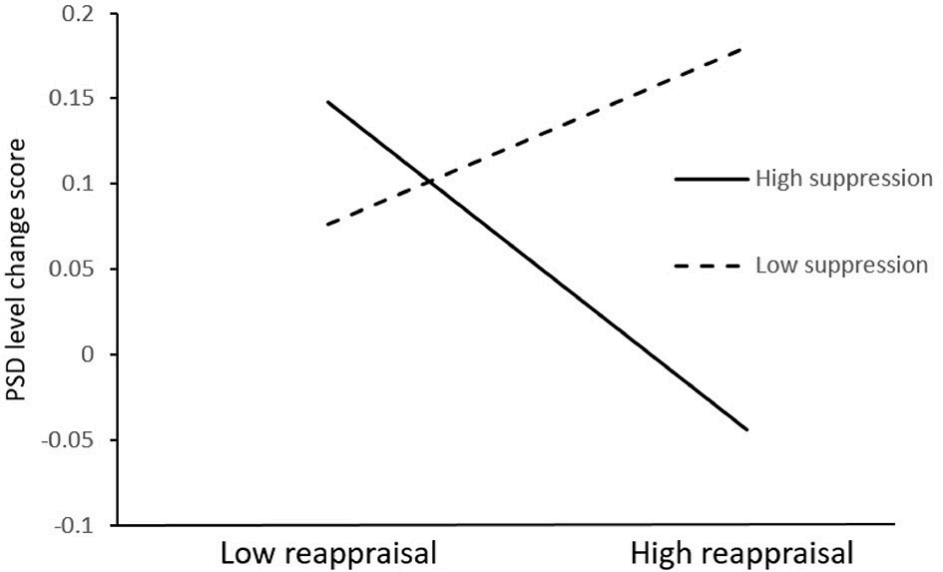
Interaction between reappraisal and suppression strategies. High and low levels correspond to 1 *SD* above or below the mean, respectively. Reappraisal strategy has a negative association with the PSD level change score only at high levels of suppression strategy.

We followed any significant or marginally significant interaction with simple slopes analyses (Aiken and West, 1991). We wanted to examine the predictions about the relationship between the reappraisal strategy and the PSD level change score, the INS change score, the DEP change score, the ANX change score, and the HOS change score in the presence of high vs. low levels of suppression strategy. Thus, we evaluated the role of the reappraisal strategy as a predictor of these five change scores at high and low levels of suppression strategy (i.e., 1 *SD* above/below the mean). At low levels of suppression strategy, the reappraisal strategy was unrelated to the PSD level change score [*t*_(98)_ = 1.64, *ns*, β = 0.192], the INS change score [*t*_(98)_ = 0.88, *ns*, β = 0.104], the ANX change score [*t*_(98)_ = 1.61, *ns*, β = 0.193], and the HOS change score [*t*_(98)_ = 1.19, *ns*, β = 0.147]. However, at high levels of suppression strategy, greater levels of reappraisal strategy was related or marginally related to lower levels of the PSD level change score [*t*_(98)_ = −2.64*, p* < 0.01, β = −0.353], the INS change score [*t*_(98)_ = −1.73, *p* = 0.09, β = −0.235], the ANX change score [*t*_(98)_ = −2.34, *p* < 0.05, β = −0.320], and the HOS change score [*t*_(98)_ = −1.73, *p* = 0.09, β = −0.244]. There was no significant relation between the reappraisal strategy and the DEP change score. As an example, Figure 1 showed the interaction between reappraisal and suppression strategies in predicting change scores of PSD level.

## Discussion

The current study examined whether individual differences in cognitive reappraisal and expressive suppression predict increase in psychopathological symptoms following acute exposure to air pollution. As hypothesized, we found that the deleterious effect of air pollution on mental health would appear even after only 1 week's acute exposure and that the effect is not limited to depression. Additionally, the present study showed that the habitual use of emotion regulation was associated with the magnitude of pollution-induced change in psychopathological symptoms. To be specific, reappraisal has a negative association with a change in positive symptom distress, anxiety, hostility, and interpersonal sensitivity only at high levels of suppression.

By prospectively examining the link between smog and increase in a repertoire of psychopathological symptoms, our findings significantly extend our knowledge of the influence of smog on psychopathology. By using regression to analyze nationwide database, Zhang et al. (2017) showed that air pollution is positively related to depression and negatively related to subjective wellbeing. Their study, however, does not shed light on whether smog is related to a wider range of psychopathology. The current study demonstrates that acute exposure to smog elevates the levels of positive symptom distress, obsessive-compulsive symptoms, interpersonal sensitivity, depression, and psychoticism. The results suggest that the effects of smog on psychological health are wide-ranging. Further longitudinal research is needed to replicate this finding and also to examine whether smog may predict the onset of clinical psychiatric disorders.

The current study found that reappraisal is negatively associated with increase in overall psychopathological symptoms as well as some dimensions of psychopathological symptoms for participants who frequently used suppression. This is consistent with Aldao and Nolen-Hoeksema's findings (2012) that adaptive strategies (e.g., reappraisal) are more adaptive when the use of maladaptive strategies (e.g., suppression and rumination) is already at high frequency. Unlike Aldao and Nolen-Hoeksema (2012) who used the composite score of several emotion regulation strategies to perform the analysis, the current work may be the first study to directly examine the interactive effects of reappraisal and suppression on psychopathology. The findings of the current study suggest that improving trait reappraisal might not have desirable effect on reducing smog-induced symptoms if the participants are already highly expressive.

Previous studies have demonstrated that emotion regulation is a transdiagnostic risk factor for psychopathology. For instance, both Aldao et al. (2010) and Webb et al. (2012) examined the effects of a wide range of emotion regulation strategies on psychopathology using meta-analysis. According to process model of emotion regulation (Gross, 1999, 2015), individuals could regulate their emotion at five points including situation selection, situation modification, attentional deployment, cognitive reappraisal and expressive suppression. It seems strange not to consider more emotion regulation strategies in this study, but in fact there are several reasons why we only pay attention to cognitive and expressive regulation of emotion. To begin with, people living in a city exposed to smog could neither switch to another city quickly nor change the situation of smog, so selecting and modifying situations might not be workable. In addition, orientating attention in smog to avoid its negative effect might be a failure because smog is so pervasive that one could hardly pay no attention to it. For these two reasons, the present study only took reappraisal and suppression into account. However, this design does not allow us to evaluate the possible roles of other emotion regulation strategies such as rumination and distraction. Thus, future studies will want to examine whether use frequencies of other emotion regulation strategies are responsible for smog-induced psychopathology.

Although ideally we should keep smog from occurring in the first place, this is not always possible. When considering the relatively long period needed to reduce pollution, certain psychological interventions would be extremely valuable for the time being. Given that cognitive reappraisal is a learnable skill (Denny and Ochsner, 2014), the current study provides a cost-effective and promising target for prevention and intervention for those higher in the habitual use of suppression in face of the smog.

Surprisingly, the current study found that suppression was negatively associated with smog-induced psychopathology (see the *Results* section in this paper for more information), which is not consistent with previous findings that suppression is maladaptive (e.g., Aldao et al., 2010). It is worth considering the two alternative accounts in future studies that may explain the discrepancy concerning the effectiveness of suppression. Indeed, suppression is generally believed to be a maladaptive strategy (Gross and John, 2003; Joormann and Stanton, 2016). However, recently it has been suggested that context might be extremely important in determining whether a strategy is adaptive or not (Aldao, 2013). For instance, suppression after exposure to traumatic scenes was shown to reduce both subjective distress during exposure and later intrusions (Dunn et al., 2009). It is possible that the nature of smog is similar to a chronic natural disaster and that suppression could be adaptive for a short while due to its contribution to the adaptation to an adverse environment. Another possible explanation is the cultural differences of emotion regulation. The current study used a Chinese sample and it has been found in previous literature that suppression is adaptive in Chinese culture, as indexed by larger declines in physiological response including skin-conductance and late positive potentials (Yuan et al., 2014a,b). We are not sure about which of the aforementioned accounts is the correct explanation for smog-induced psychopathology and it seems like an important future direction for research on air pollution and psychopathology as well as effectiveness of emotion regulation strategies.

The current study included limitations. Firstly, the current work had a relatively small sample size and was restricted to only college students. Whether the current results remain valid when it comes to other age groups and people from different social economic status remains unknown and calls upon future investigations. Young adults are generally considered to be less vulnerable when compared with old adults and children, so it is likely that smog would increase psychopathological symptoms in more vulnerable samples as well. Nevertheless, future investigations of different population would be informative since the magnitude of change in symptoms may not be the same and preventions might be more individualized when such individual differences are understood. Additionally, in this study, symptoms are measured by the Symptom Checklist-90. It is likely that the null finding in the dimension of anxiety is due to the difference of measurements and thus it would be beneficial to replicate the current findings with different measures. Moreover, due to ethical concerns we did not instruct the participants not to wear masks, and thus questions might arise as regards to whether our findings have reflected precisely the effects of air pollution on psychopathology. Similarly, previous research on air pollution and mental health rarely consider such issue (Zhang et al., 2017), but this indeed seems like a critical next step in future investigations to avoid confounding of variables. Last but not least, the pathways linking smog and psychopathology still remain unknown. There might be certain biological factors responsible for the observed effect. For instance, research has found that residents with chronically exposure to fine particulate matter also had higher concentrations of frontal pollutants, which might lead to impairments in the prefrontal cortex (Calderón-Garcidueñas et al., 2013). As prefrontal cortex is implicated in critical cognitive processes in psychopathology such as executive function (Miller, 2000; Harvey et al., 2004), such impairments might increase individual vulnerability to developing several forms of psychopathology. However, it is also possible that media coverage of the influence of pollution is playing a critical role in causing public distress. To test these possible explanations, carefully designed experiments are needed to separate the social and biological effects of the smog.

In conclusion, individual differences in the use frequencies of emotion regulation strategies following exposure to smog may predict who is likely to experience subsequent elevations in symptoms of psychopathology. Considering the fact that this link between emotion regulation and psychopathology vulnerability, it is important to understand what may contribute to individual differences in the use of emotion regulation strategies following pollution exposure. Results from the present study demonstrate that less frequent use of reappraisal in certain context may be a key factor in explaining increase in psychopathological symptoms following acute exposure to smog. Specifically, we found that lower use of reappraisal when overusing suppression predicts an increase in symptoms during a polluted period of time. This study has important implications for the individualized emotion regulation training interventions and more established preventions for people who are often exposed to smog. In other words, it will be of great importance to consider whether trainings in the use of reappraisal can be implemented only for certain participants in the face of smog, because individual differences in suppression might alter the effect of reappraisal training.

## Ethics statement

All subjects gave written informed consent in accordance with the Declaration of Helsinki prior the completion of this study. The protocol was approved by the Institutional Review Board of Tsinghua University in China.

## Author contributions

SC and JK designed the study, collected the data, performed the analysis and wrote the original manuscript. FY put forward suggestions for the study design, performed the analysis and wrote the original manuscript. KP provided valuable ideas for both the study design and manuscript contents. All the authors discussed extensively and modified the manuscript.

### Conflict of interest statement

The authors declare that the research was conducted in the absence of any commercial or financial relationships that could be construed as a potential conflict of interest.
